# An open-source zero-gradient cell hardware to improve and accelerate durability testing of PEM fuel cells

**DOI:** 10.1016/j.ohx.2023.e00495

**Published:** 2023-11-27

**Authors:** Elena Colombo, Delio Casadei, Andrea Baricci, Andrea Casalegno

**Affiliations:** Politecnico di Milano, Department of Energy, Via Lambruschini 4, Milano 20156, Italy

**Keywords:** Polymer electrolyte membrane fuel cell, Membrane electrode assembly, Durability testing, Material characterization, Zero-gradient, Hardware

## Abstract

The design of an open zero-gradient hardware is proposed in this work. The hardware is thought for characterizing single Polymer Electrolyte Membrane Fuel Cells (PEMFCs), specifically Membrane Electrode Assemblies (MEAs), with a focus on transport application. The objective of the proposed design is to minimize both operation and degradation heterogeneities over the cell active area in order to evaluate material properties only. It is indeed specifically intended to quantify the PEMFC materials performance without accounting for any interdependency between the layers of the MEA and the cell hardware design (e.g. heating/cooling system and flow field features). This is granted by combining high stoichiometry ratios of the gas reactants and limited pressure drops: they indeed keep uniform operating conditions (concentration in the gas phase, relative humidity, pressure and temperature), as verified by the electrochemical and operational characterization. With such characteristics, accurate information about both the performance and the degradation of the MEA materials can be provided. This tool is powerful for assessing the ranking in terms of performance and durability of different PEMFCs, as well as for application in the field of the materials local diagnostics.

## Highlights

• Description of an open zero-gradient hardware for fuel cell tests.
• Accurate information about both MEA performance and degradation.
• Innovative “multiple” configuration of a zero-gradient hardware.
• Comparison between standard and zero-gradient hardware.

## Graphical abstract

**Figure:**
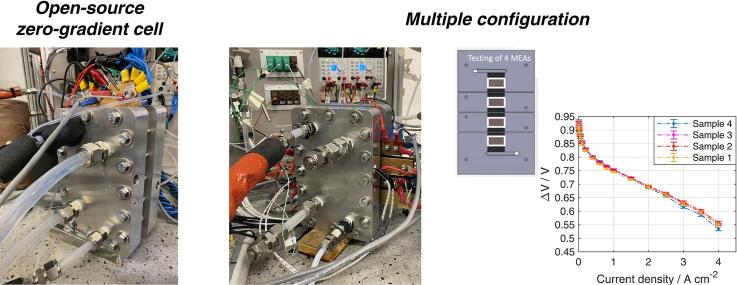

**Specifications table**.

**Table:** 

Hardware name	Polimi zero-gradient cell hardware
Subject area	Engineering and materials science
Hardware type	Differential testing hardware for electrochemical characterization of single PEM fuel cell
Closest commercial analog	Zero-gradient single cell hardware ZERO∇CELL from the European Joint Research Centre JRC (10 cm^2^ active area) and from balticFuelCells GmbH (12 cm^2^ active area)
Open source license	CERN-OHL-S
Cost of hardware	Up to 2.500 euros + VAT depending on machining cost
Source file repository	*Mendeley data:*https://doi.org/10.17632/ddzsbh34bg.1

## Hardware in context

The design of the testing hardware proposed in this work is thought for characterizing single PEM fuel cells, specifically Membrane Electrode Assemblies (MEAs) used for transport application, but it could be potentially extended to other fuel cell technologies [1], e.g*.* alkaline membrane fuel cells, high temperature PEMFCs [2].

The aim of the work is to provide the details of a “zero-gradient” configuration for testing samples from 2 cm^2^ up to 10 cm^2^ active area. The objective of the proposed design is to minimize both operation and degradation heterogeneities in order to focus on the properties of the fuel cell only. Any kind of interdependency between the MEA layers and the cell hardware design (e.g. heating/cooling system and the peculiarities of the flow field layout) must be prevented. The gradients between the inlet and the outlet regions in terms of pressure and hydrogen/oxygen concentration, usually present in commercial cells, are here avoided. We propose a gas distributor able to fulfil these requirements by adopting high reactants stoichiometry ratios while limiting as much as possible the pressure drops over the MEA active area. The stoichiometry ratios are indeed selected to be in the range 8–20: since they far exceed the standard values (around 1–2.5), they are able to keep a uniform distribution of the gases concentration. Additionally, the large stoichiometry at the cathode will result in the quicker water evacuation and less water blockage. The uniformity of the operating condition parameters (gas mole fractions, relative humidity, temperature, pressure) over the 10 cm^2^ PEMFC active area can be verified by the even local performance in the along-the-flow-field direction, proved in the present work through the “multiple” configuration of the hardware. The piece of equipment here described can be indeed used in two configurations: not only for testing a single 10 cm^2^ PEMFC, but also in a “multiple” version, which divides the flow field into four sections electrically insulated, allowing the simultaneous testing of four 2 cm^2^ samples. The results of these second configuration are described in details in the following and compared to the standard full one, to prove the consistency of the MEAs performance.

The tool is powerful both for assessing the ranking of different Catalyst Coated Membranes (CCMs) under various operating conditions and for investigating CCM degradation mechanisms. In addition, specific operating conditions that are locally encountered on automotive-size MEAs can be reproduced. Other differential hardware are available in the field, both commercial (12 cm^2^ cell hardware from balticFuelCells GmbH) and *open source* (10 cm^2^ JRC ZERO∇CELL). Details about the latter open source harmonized setup can be found in the publications by Bednarek T. and Tsotridis G. [3], [4]. What proposed in this paper is a valid and simple alternative solution that could be easily manufactured exploiting the attached drawings and used in different laboratory test benches.

## Hardware description

**Fig. 1:**
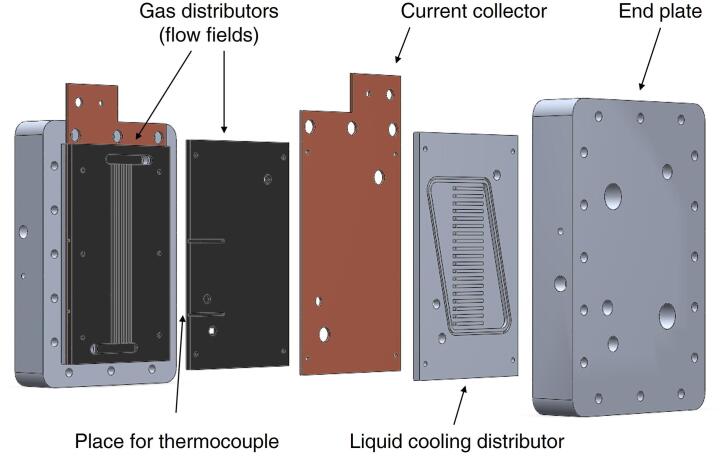
Drawing of the zero-gradient hardware and its components.

The peculiarity of the hardware, depicted in Fig. 1, consists in ensuring a uniform distribution of the operating parameters that control the MEA operation, which is desirable to obtain a stable performance. The layers included in the setup are: (*i*) two end plates; (*ii*) two liquid cooling distributors (which are optional as later explained); (*iii*) two current collectors and (*iv*) two gas distributors with parallel channels flow field geometry.

The hardware concept is based on a pattern of parallel gas channels realized in a couple of graphite plates, which reduce the pressure drops along the flow field. The hardware is operated under over stoichiometric conditions. In particular very high stoichiometries are selected both at anode (8) and at cathode (in the range 10–20) in order to avoid gradients in the concentration of the gas reactants over the MEA active area; the uniform distribution of reactants clearly leads to uniform current density too, as later shown in the results section. The reported values ensure minimum variations of the gas concentration in the along-the-flow-field direction because both the reactants consumption and the water production, due to the MEA operation, impact minimally to the extremely high flow rates. Moreover the water blockage of the channels is prevented. The reached gas velocities are more consistent with real stacks rather than those encountered in hardware setups usually adopted at the laboratory scale.

The configuration features 24 channels at cathode (0.4 mm × 0.6 mm depth/width), and 24 at anode (0.3 mm × 0.3 mm depth/width). The channel cross section is relevant because it affects the pressure drops, which must be minimized to maintain uniform conditions over the sample area. Rib width is equivalent to 0.25 mm at cathode and 0.55 mm at anode, selected to obtain sufficiently good electrical contact and secure access to gas in the region under the rib. The total width of the channels (20 mm) is imposed by the desired active area, equivalent to 10 cm^2^ in single cell configuration, and 4x2 cm^2^ in the “multiple” configuration. The total channel length is equal to 123.5 mm at cathode and 96 mm at anode. Flow field characteristics are summarized in Table 1. Note that two configurations for the cathode channels are proposed. Configuration 1 was exploited for the single cell testing (10 cm^2^ cell), adopting an air stoichiometry equal to 10. Configuration 2, which encompasses deeper channels, is useful for increasing the stoichiometry up to 20 while preventing too large pressure drops consequent to the higher gas velocities resulting from the increase of the cathodic gas flow. Configuration 2 is selected for the “multiple” setup.

**Table 1:** Flow field characteristics and geometry details of the parallel channels for the zero-gradient hardware.

	Anode	Cathode (configuration 1)	Cathode (configuration 2)
Number of channels	24	24	24
Channels depth	0.3 mm	0.4 mm	0.6 mm
Channel width	0.3 mm	0.6 mm	0.6 mm
Rib width	0.55 mm	0.25 mm	0.25 mm
Channel length	96 mm	123.5 mm	123.5 mm
Reactant stoichiometry	8	10	20

**Table 2:** CAD design files, which are available at the repository https://doi.org/10.17632/ddzsbh34bg.1. The open source licence is CERN-OHL-S.

Design file name	CAD file type
Assembly	SLDASM and STEP format
CoolingPlate_Anode	SLDPRT and STEP format
CoolingPlate_Cathode	SLDPRT and STEP format
CurrentCollector_Anode	SLDPRT and STEP format
CurrentCollector_Cathode	SLDPRT and STEP format
EndPlate_Anode	SLDPRT and STEP format
EndPlate_Cathode	SLDPRT and STEP format
FlowField_Anode	SLDPRT and STEP format
FlowField_Cathode_Channel04	SLDPRT and STEP format
FlowField_Cathode_Channel06	SLDPRT and STEP format

**Table 3:** Summary of number, cost and material type of each component of the zero-gradient hardware described in this paper.

Component	Number	Cost per unit – currency (including machining in outsourcing)	Material type
*Alternative 1 (single cell version)*			
Cooling plate	2	195 €	Metal
End plate	2	220 €	Metal
Current collector	2	190 €	Metal (no surface treatment)
Gas distributors	2	200 €	Graphite compound

*Alternative 2 (multiple version)*			
Cooling plate	2	195 €	Metal
End plate	2	220 €	Metal
Segmented Current collector	2	240 €	Metal (no surface treatment)
Segmented Gas distributors	2	250 €	Graphite compound

**Table 4:** Polarization curve operating conditions used for MEAs characterization. Adopted hydrogen/air stoichiometries are 8/10 for 10 cm^2^ zero-gradient cell while 8/20 for the 4x2 cm^2^ “multiple” zero-gradient cell. In case of oxygen, the flow rates are set equivalent to air case, causing thus an increase of stoichiometry by a factor of 4.8.

Name	T Cell °C	Dew point Cathode °C	Dew point Anode °C	RH Cathode %	RH Anode %	P_out_ Cathode kPa_abs_	P_out_ Anode kPa_abs_	*x^dry^_O2_* %
Oxygen fully humidified	80	80	80	100	100	230	250	100
Air fully humidified	80	80	80	100	100	230	250	20.9
Air dry	80	53	64	30	50	230	250	20.9

**Fig. 2:**
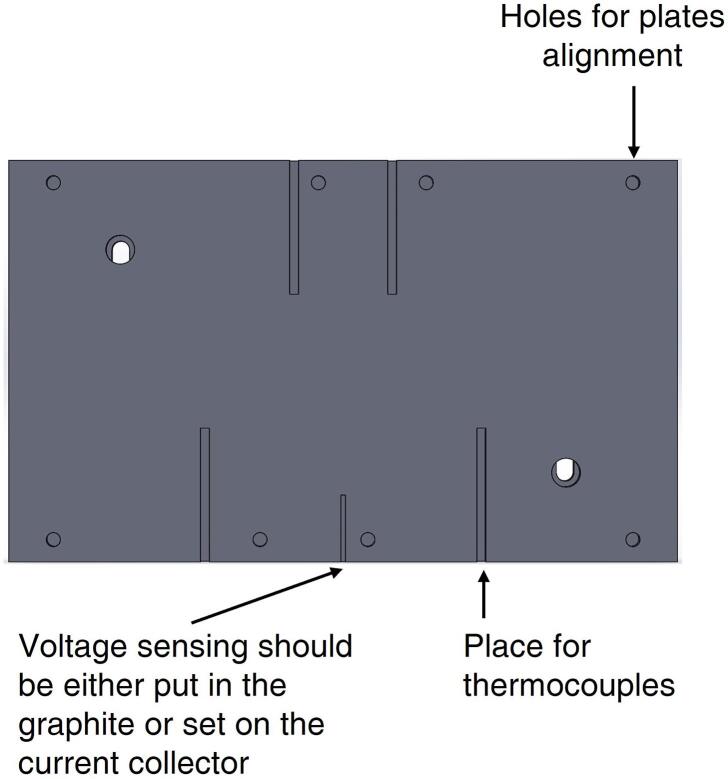
Drawing of the gas distributor plate with indications for thermocouples, voltage sensing and alignment caves position. The drawing is provided as an open-source file in the design files section.

In addition, the manifold both at inlet and outlet is designed to ensure a uniform gas distribution in all the channels. The hardware can be potentially operated both in co-flow or counter-flow configuration, thanks to its symmetry, but the results included in this work are referred to co-flow feedings. Furthermore, the hardware allows to control in an independent way the temperatures of the two sides of the flow field (anode and cathode). Temperature control is ensured by thermocouples that are inserted in the graphite gas distributors. Four hollows are placed for this purpose at both anode and cathode, as close as possible to the active area, in order to increase the measurement reliability. Caves are specifically designed at the opposite side with respect to gas channels (Fig. 2). Eight 1/8″ slots are disposed along the edge, at both the sides of the gas distributors plates. Their purpose is to hold PFA plastic (perfluoroalkoxy) centering pins, able to provide assistance in the correct assembling of the MEA and gaskets, as well as in the right alignment between the flow field and the other structural layers. The hardware ensures a versatile sensing of the cell voltage, either connecting the sensing cables to the gold-plated current collector or into dedicated caves present in the back of the graphite plate (see again Fig. 2). If proper materials are chosen for current collector and gas distributor plates, no difference in cell voltage is observed between the two measurements.

The heating of the hardware can be obtained either by a liquid-cooling system or by electric heaters inserted into the end plates, as already shown in Fig. 1. Electric heaters are in the number of two for the end plates of both anode and cathode, to ensure temperature uniformity. The liquid cooling distributor was instead designed in the heat exchanger plate, mounted between the end-plate and the current collector. This open flow field employs a simple parallel channel design, perpendicular to the direction of the gas in the graphite flow field and it aims at keeping the temperature profile as uniform as possible. It consists of 26 channels at the cathode and 20 at the anode. They are 2.5 mm deep and 2.5 mm/2.6 mm wide, at cathode/anode respectively. The open channels face the end plate in order to avoid the liquid to get in contact with the current collector plate at the other side; this solution was found to improve the electrical insulation of the cell. Sixteen M6 bolts are distributed along the end plates. The aluminum plates are designed thick enough to prevent bending and maintain a proper compression of the system. Such a compression system, based on screws and bolts, can be more easily managed in place of other commercial pneumatic configurations and does not require a continuous supply of pressurized air.

**Fig. 3:**
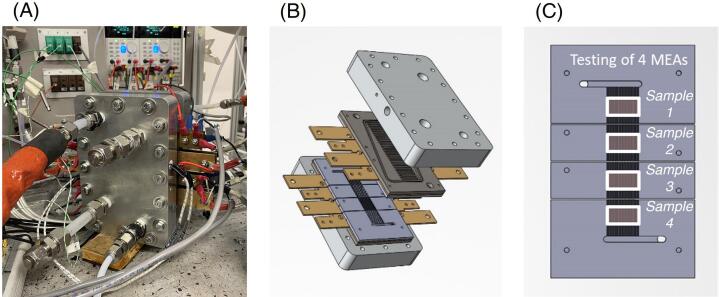
“Multiple” configuration of the zero-gradient hardware described in this paper. (A) Picture of the “multiple” configuration under cells operation; (B) CAD drawing of the hardware assembly that evidences the segmentation into four different regions, for both the gas distributors and the current collectors, which allow independent samples testing; (C) Drawing of the anode gas distributor plate with the samples location along the flow field put into evidence.

Regarding the “multiple” configuration, the gas distributors are divided into four segments, electrically insulated. They allow the simultaneous testing of a maximum of four samples of 2 cm^2^ active area each. This configuration is depicted in Fig. 3. Note that this design can be modified for testing cells with different dimensions (e.g. a lower number of cells but with a larger active area and/or a different aspect ratio). Customized features could be easily implemented since the relatively simple hardware structure here proposed assures high flexibility.

## Design files summary

- Assembly is the design file of the structure assembly;
- CoolingPlate_Anode is the design file of the anode cooling plate;
- CoolingPlate_Cathode is the design file of the cathode cooling plate;
- CurrentCollector_Anode is the design file of the anode current collector;
- CurrentCollector_Cathode is the design file of the cathode current collector;
- EndPlate_Anode is the design file of the anode end plate;
- EndPlate_Cathode is the design file of the cathode end plate;
- FlowField_Anode is the design file of the anode gas distributor plate;
- FlowField_Cathode_Channel04 is the design file of the cathode gas distributor plate with channels depth of 0.4 mm (configuration 1);
- FlowField_Cathode_Channel06 is the design file of the cathode gas distributor plate with channels depth of 0.6 mm (configuration 2).

## Bill of materials summary

- End-plate bodies are made of aluminum alloy;
- Gas distributors plates are made of a graphite compound;
- Nitrile or Viton® rubber O-rings are used for ensuring gas tight and they are commercially available. Two O-rings 3/8' are used for the current collector at the cathode side, while two 1/4' are used for the current collector at the anode side;
- Nitrile or Viton® rubber coolant circuit sealing is necessary to prevent the coolant leaks;
- Tube fittings ensure gas inlet/outlet at the end plate side: the proper size should be selected in dependance on the tube dimensions;
- The compression is obtained by a screw and bolting system. A set of 16 fixing screws (M6) is combined with the adoption of rigid gaskets. Selected rigid gaskets must have good characteristics in terms of chemical resistance and ability to withstand temperatures (up to 150 °C);
- Position pins are used for the alignment of the different plates, by means of custom-made PFA tubing;
- It is suggested to select heating cartridges with a nominal power in the range 100 W-200 W, if the cell is run on electric heating;
- Thermocouples could be placed in the number of four per sides in the gas distributor plates, care must be taken to avoid short-circuit between anode and cathode plates. The maximum diameter of the thermocouple must be 2 mm;
- The segmented configuration ensures the contemporary testing of four samples but implies slightly higher costs of manufacturing; in addition, graphite gas distributors need to be replaced more frequently because they tend to deteriorate more quickly (e.g. after two years of operation).

## Build instructions

The MEA compression is granted by combining hard gaskets to the bolted hardware system. The selection of rigid gaskets thicknesses must be done according to the different thicknesses of the MEA layers, namely diffusion media, catalyst layers and membrane.

**Fig. 4:**
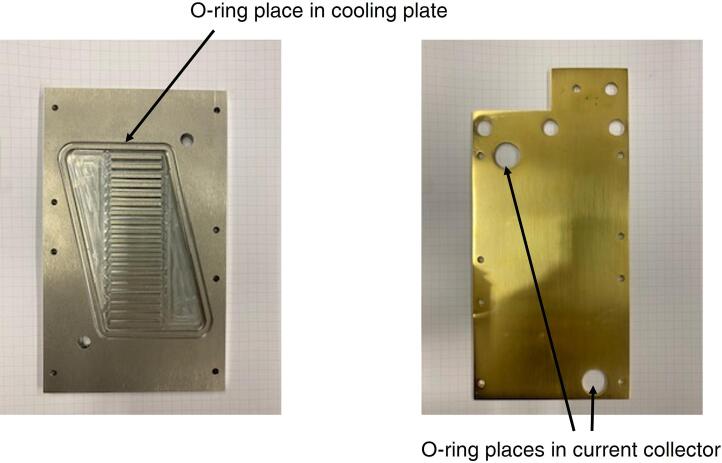
Nitrile or Viton® O-rings position into the cooling plate (if adopted) and into the golden-plate current collectors.

In addition to the assembly of the MEA, it is necessary to add proper nitrile or Viton® rubber O-rings to prevent gases or liquid leakages. O-rings must be timely substituted to maintain the setup reliability, the indication is every month. Their positioning is evidenced in Fig. 4.

**Fig. 5:**
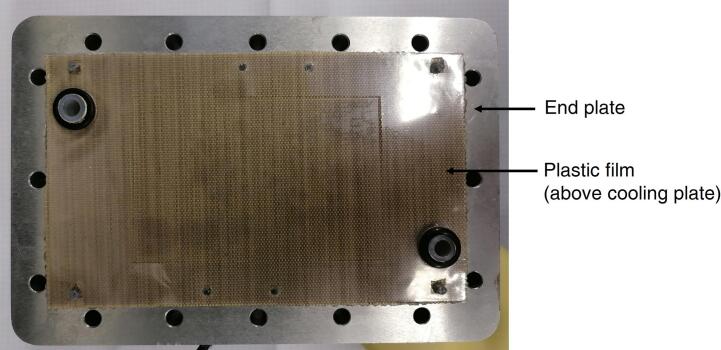
Picture of cathode end plate, cooling plate, current collector and electrically insulating film.

The different layers of the hardware must be mounted according to the sequence highlighted in Fig. 1. Note that for ensuring the electric insulation between the anode and the cathode of the cell it is necessary to place a plastic film; we normally adopt a film of glass fiber/PTFE (polytetrafluoroethylene) between the current collector and the liquid cooling plate, if present, otherwise between the current collector and the end plate. An example of the positioning of this plastic film is provided in Fig. 5. Thereafter, the sixteen M6 bolts are tightened with a torque wrench up to 8–9 Nm.

**Fig. 6:**
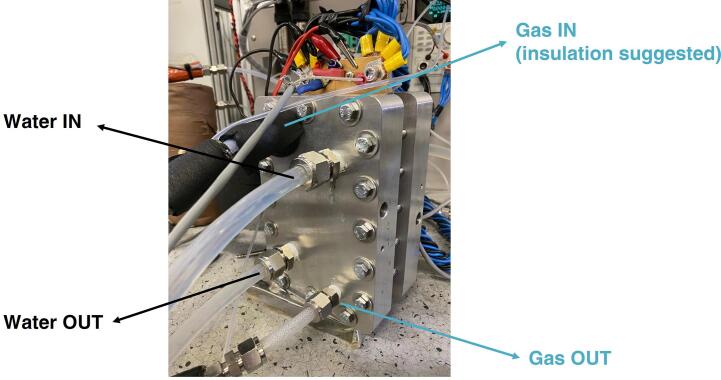
Picture of the cathode end plate with indications about the gas and water inlet/outlet positions.

**Fig. 7:**
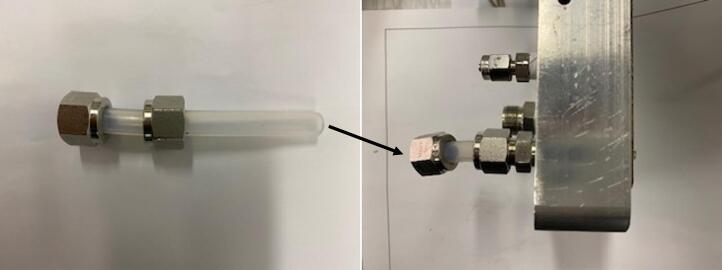
Example of PFA tube location for gas inlet/outlet. The tube passes through the different layers reaching directly the graphite gas distributor. Here the anode side is shown, but a similar solution is adopted at the cathode side too.

After having obtained the overall assembly, gases must be supplied, as well as the coolant (if used). Both the fittings can be exploited either as inlet or outlet since the structure is symmetric. An example of the provided feedings is presented in Fig. 6. The gases inlet should be thermally insulated to obtain a good performance, since insulation avoids water condensation in the humidified stream before reaching the MEA. PFA tubes are inserted at both the inlet and the outlet which avoid the gas getting in contact with the end plates and the current collectors. The tubes end directly into the gas distributor plates. Their assembly is represented in Fig. 7. The water supply for the cooling system is located on both the sides of the hardware, in the position indicated in Fig. 6; this photo shows also that the current collectors are arranged for connecting multiple sensors and the cables necessary for running the tests.

## Operation instructions

**Fig. 8:**
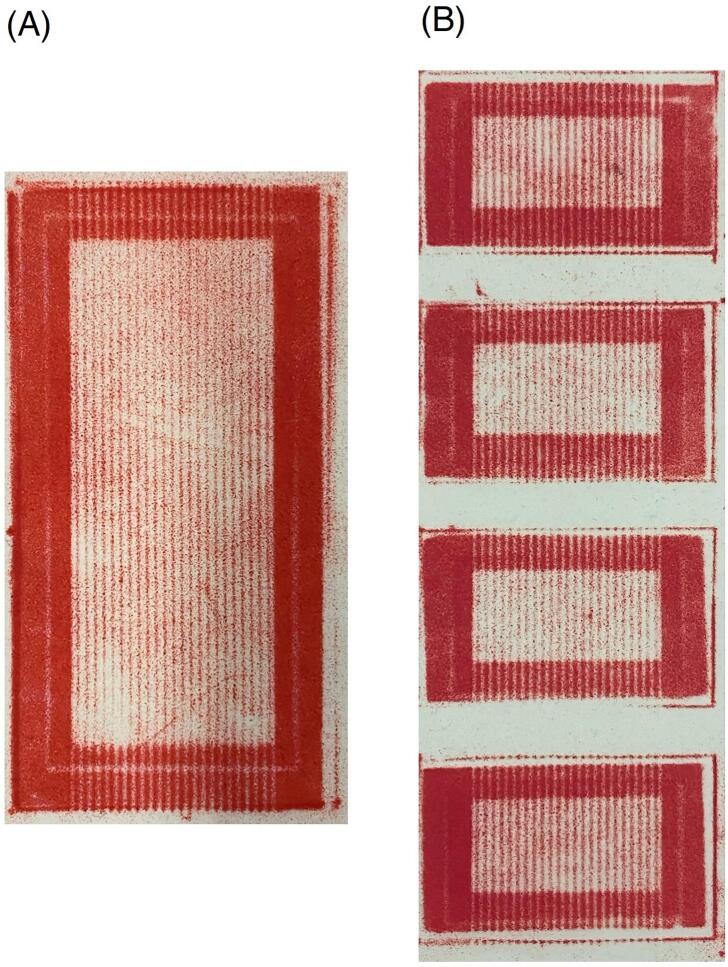
MEA compression obtained using Fujifilm LLW and applying a clamping torque of 9 Nm. Pressure distribution is quite uniform over the active area, that is the desired effect in the middle region of each sample (light red colour). The edges are instead over-compressed (dark red colour) because of the sub-gaskets; these regions are however not active. (A) Single 10 cm^2^ MEA setup; (B) “multiple” zero-gradient hardware setup. (For interpretation of the references to colour in this figure legend, the reader is referred to the web version of this article.)
b. Verify electrical insulation between the current collector plates and the liquid cooling, then with the end plates. Non perfect insulation will lead to a short circuit current between the anode and the cathode side and should be fixed by replacing the insulating plastic film placed between the current collector and the liquid cooling plates (see previous Fig. 5).
c. Once assembled, check the hardware tight by properly testing the capacity of the setup to avoid significant leakages up to an absolute pressure larger than the one used in the tests (or at least 300 kP_abs_), both at the anode and at the cathode side. Check also the absence of excessive and unwanted gas crossover through the sample, between the two setup compartments. Specific care must be dedicated to hydrogen crossover test, leak check should never be carried out using flammable gases for safety reasons. Proper protocols to check gas leakages must be defined according to the standards and codes that are beyond the objective of this work.
d. Connect all the cables and the sensors necessary for carrying out the MEA testing, verify qualified and correct connection and start the break-in/conditioning protocol of the fuel cell.

Operation must be carried out solely by trained personnel experienced with fuel cell testing and aware of safety protocols required by the application of this technology hardware, specifically (and not limited to) protocols for management of pressurized gas, electrical components and mechanical machines. Safety checks must be performed before testing and the following steps are required before starting the operation of the fuel cell:

a. It is necessary to check the proper hardware compression. It is suggested to make an evaluation through a pressure-sensitive film (e.g. Fujifilm LLW – Super Low Pressure level). An example of MEA compression obtained using Fujifilm LLW is reported in Fig. 8. The results must highlight the uniform compression of the MEA active area. Non uniform compression could evidence non-conformal manufacturing of the components or of the cell assembly.

## Validation and characterization

Results are reported for the testing of PEMFC with the hardware described in this work. The aim is to demonstrate the achievement of a uniform condition over the sample boundaries by comparing the results obtained from the single cell zero-gradient hardware with its “multiple” configuration, specifically proving consistency between the two. Several diagnostic tools are considered, including polarization curves, Electrochemical Impedance Spectroscopy (EIS) and Cyclic Voltammetry (CV). The results of the four 2 cm^2^ cells are thus compared one respect to each other, evincing minor deviations and proving the uniformity of operating conditions for all the samples. The results are also compared against the single 10 cm^2^ setup, showing, once again, a very good agreement. Results obtained from a standard triple-serpentine 25 cm^2^ hardware are finally used as a term of comparison against zero-gradient hardware to highlight the peculiarities of this latter setup.

### Materials and testing protocols

Tested MEAs in this section were obtained by assembling commercially available Pt/C Catalyst Coated Membrane (CCM) and gas diffusion layers (Freudenberg GDL H14CX483). CCM A has a catalytic loading of 0.4 mg cm^−2^/0.08 mg cm^−2^ at cathode/anode respectively. CCM B is declared as 0.5 mg cm^−2^ and 0.1 mg cm^−2^ at the two sides. Membrane thickness is 15 µm for CCM A and 18 µm for CCM B, while the electrodes are 10 µm and 15 µm thick respectively. Electrode carbon support is graphitized for both. The use of commercial materials was decided in order to mitigate differences in manufacturing, even though also in this case it is not known the standard deviation in the manufacturing reproducibility. Polarization curves conditions used in this section are summarized in Table 4.

**Fig. 9:**
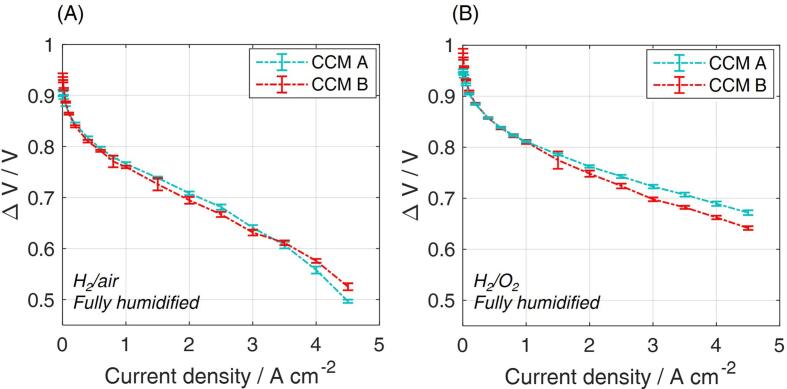
Comparison of performance of the two CCMs used for carrying out the tests. Comparison is presented on the “multiple” 2 cm^2^ configuration of the zero-gradient hardware. (A) Hydrogen/air fully humidified polarization curve (stoichiometry of 8/20) and (B) hydrogen/oxygen polarization curve, for which the flow rates have been set equivalent to those under hydrogen/air. Included standard deviation is due to the i-V protocol repetition on four independent samples for each configuration.

Each current density of the polarization curve was held for 180 s and recorded data averaged over the last 120 s, considering the progress from high to low currents. The minimum gas flow rates were selected as the equivalent at 0.5 A cm^−2^. EIS protocol was carried out three times under current control: at 1 A cm^−2^, 2 A cm^−2^ and 3 A cm^−2^, during the progress from high to low current. A total of 50 frequencies was applied, in the range from 100 mHz to 10 kHz. A comparison between the polarization curves of two CCMs analyzed in the frame of this publication is reported in Fig. 9.

## Results

### Operation under different operating conditions

**Fig. 10:**
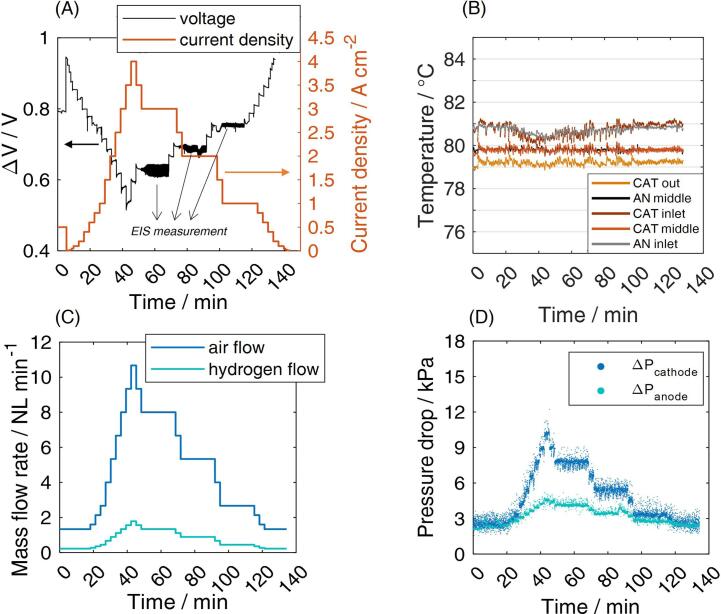
Measured variables during the polarization curve test reported as a function of time. (A) Imposed current profile during the fully humidified polarization curve and measured cell voltage (sample 3 in the 2 cm^2^ “multiple” configuration, CCM B is tested). Note that voltage oscillations at 1, 2 and 3 A cm^−2^ are due to the measurement of the impedance spectra; (B) Temperature distribution measured through the different caves available in the graphite gas distributor, where the thermocouples are inserted. Temperature control is carried out at the middle region; (C) mass flow rate of hydrogen and air provided during the polarization curve protocol; (D) pressure drops recorded during the operation of the polarization curve at both the anode and the cathode side, measured through differential pressure transducers between cell inlet and outlet.

The cell performance during the polarization curve protocol is depicted in Fig. 10A. It shows good stability (i.e. no evident fluctuations), thus proving the absence of any temporary liquid water blockage and evidencing that the hardware is able to provide accurate information. In Fig. 10C the mass flow rate supplied at anode and cathode in the “multiple” zero-gradient configuration is depicted. The evolution of the pressure drops (Fig. 10D) is proportional to the current density, namely to the supplied flows of gases. Also in this case, no significant fluctuations, which could prevent the operational stability, are observed. The largest pressure drop is at the cathode side, as expected, but the value never exceeds 10 kPa, meeting the JRC requirement [4]. Note that this quantity is measured through a differential pressure transducer placed between gas inlet and outlet, that embraces the gas manifolds, the gas fittings and part of the gas lines. The location of the pressure transducers is therefore affected by these components contributions, implying that the expected pressure drop over the only MEA active area is lower than what measured. Temperature at anode and cathode across the active area (Fig. 10B) was registered to be 80.0±1.0 °C in the whole range of current densities, fulfilling, also in this case, the JRC requirement for zero-gradient operation [4].

**Fig. 11:**
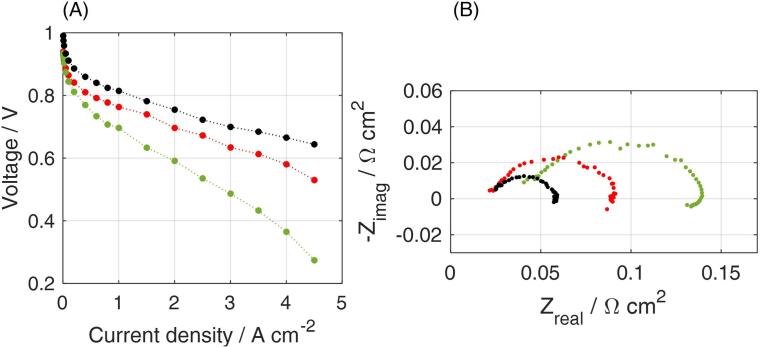
Experimental data collected for CCM B with the 2 cm^2^ zero-gradient cell under different operating conditions (see Table 2): oxygen fully humidified (black), air fully humidified (red), air dry (green). (A) polarization curves; (B) Nyquist plots obtained at 2 A cm^−2^. Table 3. (For interpretation of the references to colour in this figure legend, the reader is referred to the web version of this article.)

**Fig. 12:**
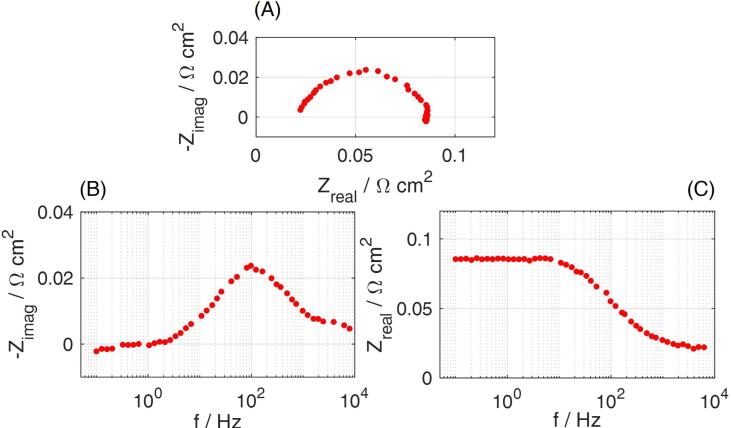
EIS at 2 A cm^−2^ for CCM B, tested in the 2 cm^2^ zero-gradient setup under air fully humidified conditions: (A) Nyquist plot: imaginary impedance as a function of the real impedance; (B) Bode plot: imaginary impedance as a function of frequency; (C) Bode plot: real impedance as a function of frequency.

Fig. 11 shows the impact of different operating conditions on CCM B, both on polarization curve and on EIS Nyquist plot. Difference between the oxygen performance (black) and air performance (red) indicates a similar ohmic contribution but an enhanced loss due to the lower oxygen concentration in the air case. The dry case (green) is instead responsible for larger ohmic losses due to the higher membrane resistance and higher ionic resistance into the cathode catalyst layer. These conclusions are drawn based on the analysis of EIS spectra in Fig. 11B where the High Frequency resistance, i.e*.* intercept with real axis at the highest frequencies, and the 45° linear branch at high frequency (left side of the chart) are both found to increase under low relative humidity. For completeness, Nyquist and Bode diagrams are reported for the air fully humidified operating conditions in Fig. 12.

### Full and “multiple” zero-gradient configurations: A comparison

**Fig. 13:**
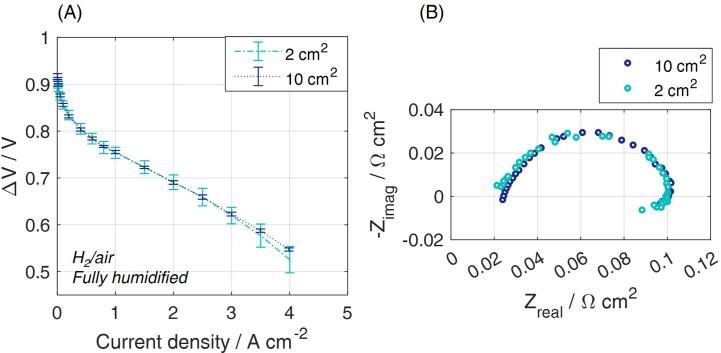
Comparison of performance in the two proposed versions of the zero-gradient hardware. (A) Fully humidified polarization curve recorded in the full 10 cm^2^ configuration (stoichiometry of 8/10) and in the “multiple” 2 cm^2^ configuration on CCM A (stoichiometry of 8/20). Included standard deviation is due to the protocol repetition for six independent samples for each configuration; (B) Nyquist plot obtained at the current density of 1 A cm^−2^ at fully humidified conditions, for the frequency range 100 mHz-10 kHz.

**Fig. 14:**
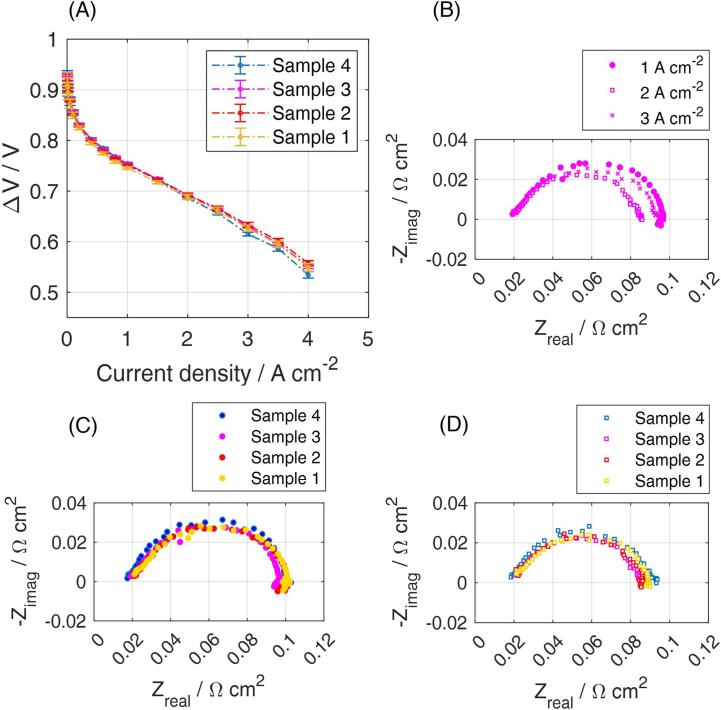
Comparison of the four 2 cm^2^ samples of CCM B tested in the multiple cell configuration of the zero-gradient hardware, placed according to the scheme in Fig. 3.C under air fully humidified conditions. (A) Polarization curves. Standard deviation is calculated for four different CCMs tested at each sample position; (B) Nyquist plot recorded at different current densities, as indicated in the legend, for the sample in position 3; (C) Nyquist plot recorded for the different samples positions at 1 A cm^−2^; (D) Nyquist plot recorded for the different samples positions at 2 A cm^−2^.

CCM A was selected for comparing 10 cm^2^ and 2 cm^2^ cell performance. The polarization curves obtained from the current-descending scan, both in the full and the “multiple” setup, are included in Fig. 13A. The deviation is calculated according to the results repeatability in case of six independent MEA samples, used for each configuration. A good consistency is highlighted in spite of a possible more relevant edges effect of the 2 cm^2^ MEA. Not only, but a good consistency is also obtained for the impedance spectra reported in Fig. 13B, over the whole frequency range. Furthermore, the results of the testing campaign on the 2 cm^2^ cells have been evaluated with respect to the position of the MEA in the flow field. Performance of samples in positions 1, 2, 3 and 4, according to the scheme of Fig. 3C, are compared in Fig. 14. On one hand, the good repeatability of the results for each specific position is proved. On the other hand, deviations among positions 1–4 are minor. Up to 2.5 A cm^−2^, the results agreement is extremely high (deviations < ±4 mV, comparable with the uncertainty measurement). At currents as high as 4 A cm^−2^, sample 4 performance is lower and deviation is slightly larger ±10 mV. Fig. 14C and D prove that also impedance spectra agree over the whole range of frequencies.

The Nyquist plot included in Fig. 14B clarified that from 1 A cm^−2^ to 2 A cm^−2^ the contribution of the Oxygen Reduction Reaction (ORR) decreases, resulting in a smaller arch. The main arc in the Nyquist plot accounts indeed for the charge transfer of the ORR, occurring in the cathode catalyst layer (R_CT_), which depends on the electrode potential: the diameter of the loop decreases while decreasing the cell voltage, due to the enhanced driving force of the higher reaction overpotentials. The range between 1 and 2 A cm^−2^ corresponds to the ohmic region of the polarization curve. The Nyquist plot at 3 A cm^−2^ would correspond to a further reduction of the ORR contribution but shows a possible slight increase of the oxygen transport losses due to oxygen diffusion. It is indeed observed a small increase of the total cell resistance between 2 A cm^−2^ and 3 A cm^−2^, where the total cell resistance is defined as the intercept of the plot with the x-axis at the lowest frequencies (i.e*.* right side of the chart). Mass transport limitations are however clearly rising under hydrogen/air and fully humidified operating conditions for current densities above 3.5 A cm^−2^, as seen in the polarization curve of Fig. 11A.

**Fig. 15:**
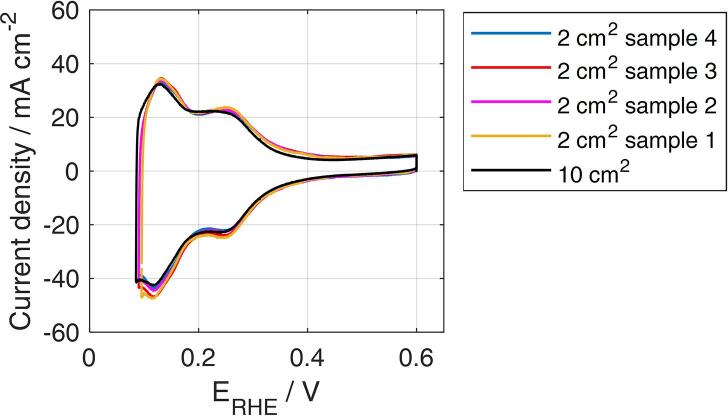
Cyclic Voltammetry recorded as fourth scan between OCV and 0.6 V. H_2_-rich anode is used as a dynamic reference electrode and a scan rate of 100 mV s^−1^ is applied, at 30 °C and under fully humidified H_2_/N_2_ flows, both equivalent to 0.1 NL min^−1^. Results are reported for the “multiple” configuration in the different positions (coloured) and the full configuration (black). CCM B results are here included.

Reproducibility of results is finally verified on cyclic voltammetry, used for quantifying the catalyst electrochemically active surface area (Fig. 15). Minor deviations are between the 2 cm^2^ samples in positions 1–4 and with respect to the single 10 cm^2^ PEMFC. The general congruity of performance of samples 1–4 indicates that the zero-gradient setup is indeed working under uniform conditions over the active area of all the MEAs.

### Zero-gradient and typical cell setups: a comparison

A standard 25 cm^2^ single cell setup based on a triple serpentine flow field with 0.85 mm × 0.85 mm channels (named standard SC in the following) is used as a term of comparison for evidencing the peculiarities of the zero-gradient hardware and help in comparing the outputs under different setups [5]. The specifications of this cell can be found in previous works [6], [7], [8].

**Fig. 16:**
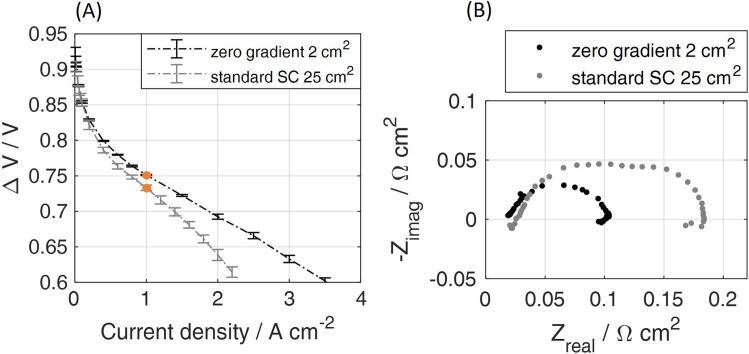
Comparison for CCM B between the zero-gradient and the standard single cell (SC) hardware. (A) Air fully humidified polarization curve. For the standard SC, imposed operating conditions are the same as included in Table 2, while stoichiometries are 2/4 for anode and cathode respectively; (B) EIS reported in the Nyquist plane and recorded at 1 A cm^−2^ for the standard SC setup, compared against the result in the zero-gradient configuration.

The fully humidified polarization curve of the standard SC is reported in Fig. 16A, alongside the zero-gradient performance. It is immediately evident the more extended ΔV-i linear behaviour of the zero-gradient setup, in a significantly wider range of current densities (between 1 A cm^−2^ and 3.5 A cm^−2^, against the deviations already visible at 1 A cm^−2^ for the standard SC). The MEA performance could be tested up to high current densities, evidencing its intrinsic properties and avoiding pronounced mass transport limitations due to non-uniform gas distribution and liquid water effects that are related to the flow field geometry. The different role of mass transport phenomena in the two setups is clear in the EIS spectrum, reported as Nyquist plots in Fig. 16B. The comparison indicates that the HFR is subjected only to minor changes (being slightly lower in zero-gradient compared to SC hardware), while the total resistance is instead significantly higher for the standard SC, almost doubled: this is attributed to transport limitations effects that are mainly due to the lower stoichiometry allowed in the standard hardware configuration but also testing hardware-specific (e.g. hardware plates resistivity, channels design).

### CRediT authorship contribution statement

**Elena Colombo:** Methodology, Conceptualization, Investigation, Formal analysis, Data curation, Writing – original draft. **Delio Casadei:** Investigation, Data curation. **Andrea Baricci:** Methodology, Conceptualization, Writing – review & editing. **Andrea Casalegno:** Supervision, Project administration.

## Declaration of competing interest

The authors declare that they have no known competing financial interests or personal relationships that could have appeared to influence the work reported in this paper.
